# Four-dimensional flow cardiac magnetic resonance assessment of left ventricular diastolic function

**DOI:** 10.3389/fcvm.2022.866131

**Published:** 2022-07-22

**Authors:** Zakariye Ashkir, Saul Myerson, Stefan Neubauer, Carl-Johan Carlhäll, Tino Ebbers, Betty Raman

**Affiliations:** ^1^Oxford Centre for Clinical Magnetic Resonance Research (OCMR), Division of Cardiovascular Medicine, Radcliffe Department of Medicine, University of Oxford, Oxford, United Kingdom; ^2^Division of Diagnostics and Specialist Medicine, Department of Health, Medicine and Caring Sciences, Linköping University, Linköping, Sweden; ^3^Center for Medical Image Science and Visualization (CMIV), Linköping University, Linköping, Sweden; ^4^Department of Clinical Physiology in Linköping, Department of Health, Medicine and Caring Sciences, Linköping University, Linköping, Sweden

**Keywords:** diastolic function, heart failure, 4D flow cardiac MR, kinetic energy, flow components, vortex

## Abstract

Left ventricular diastolic dysfunction is a major cause of heart failure and carries a poor prognosis. Assessment of left ventricular diastolic function however remains challenging for both echocardiography and conventional phase contrast cardiac magnetic resonance. Amongst other limitations, both are restricted to measuring velocity in a single direction or plane, thereby compromising their ability to capture complex diastolic hemodynamics in health and disease. Time-resolved three-dimensional phase contrast cardiac magnetic resonance imaging with three-directional velocity encoding known as ‘4D flow CMR’ is an emerging technology which allows retrospective measurement of velocity and by extension flow at any point in the acquired 3D data volume. With 4D flow CMR, complex aspects of blood flow and ventricular function can be studied throughout the cardiac cycle. 4D flow CMR can facilitate the visualization of functional blood flow components and flow vortices as well as the quantification of novel hemodynamic and functional parameters such as kinetic energy, relative pressure, energy loss and vorticity. In this review, we examine key concepts and novel markers of diastolic function obtained by flow pattern analysis using 4D flow CMR. We consolidate the existing evidence base to highlight the strengths and limitations of 4D flow CMR techniques in the surveillance and diagnosis of left ventricular diastolic dysfunction.

## Background

Left ventricular diastolic dysfunction (LVDD) is characterized by increased ventricular stiffening coupled with impaired myocardial relaxation. Although these processes occur naturally with aging, they are accelerated and worsened in many cardiovascular conditions.

Left ventricular diastolic dysfunction is a major cause of heart failure syndrome in the form of heart failure with preserved ejection fraction (HFpEF), which carries a poor prognosis (1, 2). Due to persistently raised intra chamber pressures and remodeling, it can lead to atrial fibrillation (3), pulmonary hypertension (4) and right ventricular failure (5). As an early marker of cardiac impairment and one that often precedes clinical manifestation of disease, the timely diagnosis of LVDD is important for patient management.

The diagnosis of HFpEF however remains challenging because of a lack of consensus on the assessment of left ventricular (LV) diastolic function, and because our understanding of complex diastolic hemodynamics is incomplete. Furthermore, many patients are incorrectly diagnosed because the symptoms of heart failure may overlap with manifestations of other conditions (e.g., respiratory illness). As a result, there is high need for good imaging assessment of LVDD, to provide a positive reason for diagnosing this condition, and avoid overreliance on symptoms to make the diagnosis of HFpEF.

In this review, we examine a potentially useful diagnostic tool in diastolic heart failure—diastolic left ventricular blood flow assessment with four-dimensional flow cardiac magnetic resonance (4D flow CMR). This is an emerging technology which has the potential not only to improve early detection of LVDD, but also to expand our understanding of diastolic hemodynamics and ventricular function by going beyond the conventional parameters of LV diastolic function.

## Challenges in conventional assessment of left ventricular diastolic function

Echocardiography has traditionally been the modality of choice for non-invasive assessment of LV diastolic function. Current American Society of Echocardiography (ASE) and European Association of Cardiovascular imaging (EACVI) guidelines (6) recommend a combination of myocardial and blood flow velocities to diagnose LVDD, using measurements of trans-mitral, mitral annular and tricuspid regurgitation velocities. The presence of atrial dilatation and abnormal pulmonary venous flow can further support the diagnosis of LVDD. Newer techniques such as strain rate imaging and three-dimensional (3D) echo can also provide additional information on LV diastolic function. Although echocardiography is well-validated for LVDD diagnosis and has obvious practical benefits, measurement accuracy is vulnerable to inter-operator variability and suboptimal acoustic windows (e.g., due to patient body habitus or comorbidities). Furthermore, the accuracy of one of the important parameters for assessing LVDD (E/e′) is very dependent on good alignment of the Doppler beam with the long axis of the left ventricular walls and mitral valve, which is often difficult to achieve. Even when echocardiographic measurements are of sufficient quality and accuracy, conflicting results in LV diastolic function parameters (some within normal range, whilst others are pathological) and a large ‘intermediate’ range of values (between normal and abnormal) contribute to a large proportion of patients being classified as having ‘indeterminate’ diastolic function according to current EACVI/ASE algorithms (7).

## Cardiac magnetic resonance potential for assessing left ventricular diastolic function

Cardiac magnetic resonance (CMR) is recognized as the gold standard modality for measurement of cardiac chamber dimensions, volumes, and systolic function, but it can also be used to assess LV diastolic function. Two-dimensional phase contrast CMR (referred to as “2D PC-CMR” for the purpose of this review) measurements of trans-mitral, mitral annular and pulmonary venous velocities, as well as flow, enable diagnosis and assessment of LVDD (8). Diastolic function may also be assessed using 3D volumetric assessment of diastolic LV filling (9) and by strain imaging (10). Combined with advanced techniques such as tissue characterization, the sensitivity of CMR to diagnose important underlying etiologies such as hypertrophic cardiomyopathy (11) and cardiac amyloidosis is enhanced (12). Despite these complimentary approaches to echocardiography, there is currently no universally accepted approach for assessing LV diastolic function on CMR. Velocity/flow measurement using both echocardiography and 2D-PC CMR are further constrained to a single direction and plane. This results in an inability to fully characterize blood flow dynamics, since blood flow is a three-dimensional phenomenon in constant motion, with different velocities, directions, and acceleration at different times during the cardiac cycle.

## Four-dimensional flow cardiac magnetic resonance imaging

Four-dimensional flow cardiac MRI (4D flow CMR) involves the acquisition of time-resolved three-dimensional phase contrast imaging with three-directional velocity encoding (13, 14). With the time resolved 3D velocity data obtained, complex aspects of blood flow and myocardial function can be studied throughout the cardiac cycle.

Using 4D flow CMR, the velocity vector is measured everywhere in a 3D volume over the cardiac cycle allowing the retrospective creation of velocity maps, and by extension flow, at any point in the acquired 3D data volume (14, 15). 4D flow CMR can therefore facilitate the quantification of complex hemodynamic and functional parameters such as kinetic energy, relative pressure, energy loss and vorticity.

In the past decade with greater experience and advances in acquisition techniques, post processing and analysis, 4D flow CMR has become more feasible and clinically relevant as summarized by Crandon et al. (16) and Demirkiran et al. (17). Image acquisition can be performed using free breathing techniques, and 4D flow CMR has shown good reproducibility (18, 19) with proven reliability when compared to 2D PC-CMR and echo (20–22).

In terms of assessment of LV diastolic function, 4D flow techniques such as retrospective valve tracking, can be used to accurately measure conventional flow parameters of LV diastolic function such as mitral inflow velocities with good correlation with echocardiographic measurements (21, 23, 24). More recently, novel and more complex flow pattern-based parameters have also been used to study diastolic flow hemodynamics. This review is the first to focus on diastolic flow pattern analysis using 4D flow CMR and its potential clinical application.

## Assessment of left ventricular diastolic function with four-dimensional cardiac flow pattern analysis

**FIGURE 1:**
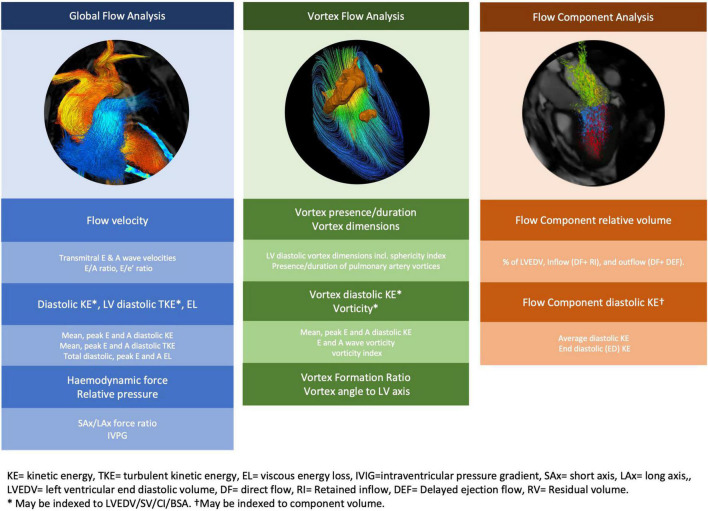
Novel 4D flow CMR parameters of left ventricular diastolic function. Novel 4D flow CMR parameters are obtained from analysis of global flow, vortex flow and/or functional flow components.

Many different flow pattern-based parameters have been explored by 4D flow CMR studies for more in-depth evaluation of LV diastolic flow hemodynamics and diastolic function (Figure 1). Additionally, as 4D flow CMR enables voxel wise mapping of many of these novel parameters (e.g., kinetic energy and vorticity), they can be used to analyze localized flow patterns allowing focused study of specific regions such as the left ventricular outflow tract (LVOT) (25, 26). A summary of the studies of LV flow patterns divided based on analysis of global flow, vortex flow or (functional) component flow provides a useful reference framework (summarized in Tables 1–6).

**TABLE 1:** Four-dimensional flow CMR studies assessing left ventricular diastolic function using global flow diastolic KE.

References	*N*	Disease/topic	4D diastolic parameters	Comparison vs. conventional diastolic parameters	Relevant findings
**Healthy controls, aging and athletes**					
Crandon et al. (27)	53 controls	Aging and LVDD	LV diastolic KE*, LV peak E and A wave KE*, KE E/A ratio*	CMR derived E, A velocities and E/A ratio. Direct comparison showed a significant positive correlation with KE E/A ratio.	Aging associated with changes in LV diastolic KE parameters: decline in peak E-wave KE and increase in peak A-wave KE. Diastolic KE assessment may be more reliable than conventional diastolic parameters.
Steding-Ehrenborg et al. (33)	14 athletes 14 controls	Athletes vs. normal	Peak E and A wave KE	−	Athletes have higher LV and RV early diastolic peak KE. LV mass is the main determinant of LV diastolic KE.
Carlsson et al. (25)	9 controls	Normal blood flow	LV peak E and A wave diastolic KE	−	Early diastole KE greater in LV than RV, suggesting LV early filling more dependent on suction. Mean KE related to volume and similar in LV and RV.
**Cardiovascular disease**					
Riva et al. (35)	10 HF patients (ischemic) 10 AL cardiac amyloidosis patients 8 controls	HF (ischemic) Cardiac amyloidosis	Mean systolic and diastolic KE*, peak E and A wave KE, viscous energy loss, hemodynamic force and flow component volumes	−	HF associated with reduced mean systolic and diastolic KE and peak E wave KE. HF patients also had a significant reduction in base to apex hemodynamic force component. Cardiac amyloidosis was associated with reduced peak E wave KE.
Garg et al. (34)	36 MI patients + LV thrombus 34 MI patients – LV thrombus 40 controls	Post MI	Peak E and A wave KE, regional average TD	CMR derived E, A velocities and E/A ratio. No direct association made with 4D diastolic parameters.	Significant reduction in peak E wave KE in MI patients. Significant drop in A wave KE from mid ventricle to apex in MI patients with LVT. MI patients with LVT also had delayed peak A wave KE.
Garg et al. (32)	48 MI patients 20 controls	Post MI	Mean diastolic KE*, Peak E and A wave KE*, in-plane KE*, TD.	−	LV impairment post MI associated with reduced peak E wave diastolic KE. Infarct size associated with increased in-plane (pathological) LV blood flow KE.
Wong et al. (30)	10 HF patients 35 controls incl. children	Aging vs. HF	Peak E and A wave KE*, mid diastolic (diastasis) KE*	−	Peak diastolic KE progressively decreased with age, whereas systolic peaks remained constant. Peak diastolic KE in the oldest subjects comparable to those with LV dysfunction.
Kanski et al. (31)	29 HF patients 12 controls	HF (mixed)	Peak E and A wave KE*, mean diastolic KE*	CMR derived E, A velocities, E/A ratio, Deceleration time, LAV/BSA, and pulmonary venous flow profile. No results shared or direct made with 4D diastolic parameters.	No difference in mean diastolic KE. In patients, a smaller fraction of diastolic KE observed inside vortex. Determinants of diastolic KE were LVM and PFR.
Al-Wakeel et al. (36)	10 MR patients 10 controls	Pre vs. post MV surgery	Peak E and A wave KE*, Mean diastolic KE	Echocardiography and CMR derived E, A velocities and E/A ratio. Direct comparison showed significant correlation of E/A ratio with KE E/A ratio but only in postoperative patient cohort.	Along with a reduction of LV end diastolic, end-systolic end stroke volume, mean, systolic, and early diastolic KE decrease significantly after MV surgery. However late diastolic KE remained high.

BSA, body surface area; EDV, end diastolic volume; HF, heart failure; KE, kinetic energy; LAV, left atrial volume; LV, left ventricle; LVDD, left ventricular diastolic dysfunction LVM, left ventricular mass; LVT, left ventricular thrombus; MI, myocardial infarction; MR, mitral regurgitation; MV, mitral valve; PFR, peak filling rate; SV, stroke volume; TD, time difference to peak E wave from base to apex. *Indexed to LVEDV/SV.

**TABLE 2:** Four-dimensional flow CMR studies assessing left ventricular diastolic function using other novel 4D global flow parameters.

Reference	*N*	Disease/topic	4D diastolic parameters	Comparison vs. conventional diastolic parameters	Relevant findings
**Healthy controls**					
Casas et al. (54)	9 controls	Dobutamine stress	Contraction rate constant, relaxation constant, elastance diastolic time constant	−	Stress resulted in differences in load-independent parameters: contraction rate constant, relaxation constant and elastance diastolic time constant.
Eriksson et al. (44)	12 controls	Relative Pressure	Relative pressure	−	Relative pressure was heterogeneous in the LV, with the main pressure difference along the basal-apical axis.
**Cardiovascular disease**					
Arvidsson et al. (51)	39 HF patients with LBBB 31 controls	HF (mixed etiology) Dyssynchrony	Hemodynamic force, diastolic transverse and longitudinal force ratios	−	Patients with dyssynchrony exhibited increased transverse forces. Diastolic force ratio was able to separate controls from patients.
Elbaz et al. (41)	32 corrected AVSD patients 30 controls	Energy loss	Mean and peak E and A wave EL*, mean and peak E and A wave diastolic KE*	CMR derived E, A velocities and E/A ratio. Direct comparison showed moderate correlation between E/A ratio and Energy Loss E/A ratio	Abnormal diastolic vortex formation was associated with increased viscous energy loss.
Eriksson et al. (50)	18 HF patients	HF (mixed etiology) Dyssynchrony	Hemodynamic force, Sax/Lax-max force ratio	−	LV filling forces more orthogonal to the main LV flow direction in LBBB during early diastole. The greater the conduction abnormality the greater the discordance of LV filling force with predominant LV flow direction.
Eriksson et al. (55)	10 DCM patients 10 controls	HF (DCM)	Hemodynamic force, SAx/LAx force ratio	−	SAx/LAx ratio significantly larger in DCM patients compared to healthy subjects. DCM patients had forces that were more heterogeneous in their direction and magnitude during diastole.
Zajac et al. (42)	9 DCM patients 11 controls	HF (DCM)	LV diastolic TKE, LV peak E and A wave TKE	Echocardiography derived E, A velocities. Direct comparison showed correlation with peak late (A) velocity.	Late diastolic turbulent kinetic energy (TKE) was higher in DCM patients with diastolic dysfunction compared to control.

AVSD, atrioventricular septal defect; DCM, dilated cardiomyopathy; EL, energy loss; HF, heart failure; KE, kinetic energy; LBBB, left bundle branch block; LV, left ventricle; SAx, short axis; LAx, long axis; TKE, turbulent kinetic energy. *Indexed to LVEDV/SV.

**TABLE 3:** Four-dimensional flow CMR studies assessing left ventricular diastolic function using vortex flow analysis.

Study Year	*N*	Disease/topic	Diastolic vortex parameters	Comparison vs. conventional diastolic parameters	Relevant findings
**Healthy controls, aging and athletes**					
Nakaji et al. (26)	19 controls	Normal physiology	EL, ELI, diastolic KE	−	Large end-diastolic vortices with low EL observed which facilitated blood flow toward the aortic valve.
Rutkowski et al. (69)	39 controls	Sex differences	Diastolic kinetic energy, strain, vorticity, vorticity index (SV)	−	Women have higher diastolic vorticity and strain rates and lower blood flow KE.
Steding-Ehrenborg et al. (33)	14 athletes 14 controls	Athletes vs. normal	vortex diastolic KE, vortex area and volume (not reported)	−	70% of diastolic KE found inside LV diastolic vortex. Positive physiological remodeling preserves vortex formation and diastolic KE.
Elbaz et al. (62)	24 controls	Normal physiology	Vortex circularity index, vortex orientation	CMR derived E, A velocities and E/A ratio. No direct comparison made with 4D diastolic parameters*	Differences observed between early and late diastolic vortices in terms of vortex shape, location of vortex core. Vortex shape correlated with mitral inflow shape.
Foll et al. (57) 2013	24 controls	Age and sex differences	Vortex area, vortex peak velocity, vortex duration	−	Vortex number, size and velocities varied with age, gender, blood pressure, LVEDV and ejection fraction.
Kim (56)	26 controls	Normal physiology	Vortex radius, vortex angular velocity, vortex kinetic energy	CMR derived E, A velocities. No direct comparison made with 4D diastolic parameters	Early confirmation study of diastolic vortex formation and its close relationship with the mitral valve.
**Cardiovascular disease**					
Krauter et al. (63)	10 IHD patients 10 controls	Automated analysis	Vortex ring volume, circularity index, angle to LV long axis, vorticity, vortex ring KE	CMR derived E, A velocities. Direct comparison showed a significant correlation with vorticity and vortex ring KE.	Vorticity and kinetic energy of the early diastole vortex was significantly greater in controls compared to IHD patients and correlated strongly with trans-mitral E velocities.
Schäfer et al. (68)	16 COPD patients 10 controls	LVDD in COPD	Vorticity	Echocardiography derived E, A velocities and E/A ratio. Direct comparison made with 4D RV, not LV diastolic parameters.	Diastolic vorticity is reduced in patients with mild-to-moderate COPD with no or mild signs of LVDD on echocardiography. Reduced diastolic vorticity in COPD patients is a sensitive and early marker of LVDD. LV E phase vorticity correlated with 6MWT.
Elbaz et al. (41)	32 corrected AVSD patients 30 controls	Mitral valvulopathy	Vortex formation, EL, diastolic KE	CMR derived E, A velocities and E/A ratio. Direct comparison showed an only moderate correlation between E/A ratio and EL E/A ratio	Abnormal diastolic vortex formation was associated with increased viscous energy loss.
Suwa et al. (65)	21 controls 14 HF patients	HF (mixed etiology)	Vortex area, distance to vortex core,	−	In patients with severe LV systolic dysfunction and dilatation, diastolic vortices were more apically located, larger and more spherical.
Schäfer et al. (67)	13 PH patients 10 controls	Vorticity in pulmonary hypertension	Vorticity	Echocardiography derived E, A velocities and E/A ratio. Direct comparison showed E and A wave vorticity correlated with multiple diastolic parameters incl. E/A ratio.	Early diastolic (E wave) vorticity was significantly reduced in PH patients, and correlated with LVDD markers including E, E/A and e′.
Töger et al. (70)	23 controls 23 HF patients	LV diastolic function	Vortex formation ratio, mixing ratio, vortex volume, vortex volume/LV volume in diastasis	CMR derived E, A velocities, E/A ratio, Deceleration time, LAV/BSA, and pulmonary venous flow profile. Direct comparison found no significant correlations.	Heart failure patients had a greater mixing ratio (mixing of inflowing and surrounding fluid in the vortex) which moderately correlated with peak diastolic inflow velocity.
Kanski et al. (31)	29 HF patients 12 controls	HF	vortex ring size, vortex diastolic KE	CMR derived E, A velocities, E/A ratio, Deceleration time, LAV/BSA, and pulmonary venous flow profile. No results shared or direct compared made with 4D diastolic parameters*	Heart failure patients had a smaller fraction of diastolic KE inside the vortex ring compared to controls.

AVSD, atrioventricular septal defect; BSA, body surface area; COPD, chronic obstructive pulmonary disease; EL, energy loss; ELI, energy loss index; HF, heart failure; IHD, ischaemic heart disease; KE, kinetic energy; LAV, left atrial volume; LV, left ventricle; LVEDV, left ventricular end diastolic volume; LVDD, left ventricular diastolic dysfunction; PH, pulmonary hypertension; 6MWT, 6-minute walk test.

**TABLE 4:** Flow components as percentage of LVEDV in healthy controls.

	*N*	Direct Flow	Retained Inflow	Delayed Ejection Flow	Residual Volume
Bolger et al. (71)	17	**21** ± 6%	**27** ± 8%	**27** ± 6%	**24** ± 12%
Eriksson et al. (28)	6	**35** ± 6%	**17** ± 4%	**15** ± 3%	**33** ± 4%
Eriksson et al. (72)	12	**37** ± 5%	**17** ± 4%	**16** ± 3%	**30** ± 5%
Eriksson et al. (74)	10	**38** ± 5%	**17** ± 3%	**16** ± 3%	**29** ± 5%
Svalbring et al. (75)	10	**42** ± 8%	**19** ± 2%	**17** ± 3%	**23** ± 5%
Stoll et al. (76)	45	**38** ± 4%	**16** ± 4%	**16** ± 3%	**30** ± 4%
Corrado et al. (73)	10	**58** ± 11%	**15** ± 6%	**16** ± 6%	**7** ± 6%
Sundin et al. (77)	12	**36** ± 6%	**20** ± 3%	**17** ± 3%	**27** ± 4%

**TABLE 5:** Four-dimensional flow CMR studies assessing eft ventricular diastolic function using flow component analysis.

Reference	*N*	Disease/subject	4D diastolic parameters	Comparison vs. conventional diastolic parameters	Relevant findings
**Healthy controls**					
Sundin et al. (77)	12 controls	Dobutamine stress	Flow component **volumes** Flow component diastolic KE*	−	Improved flow efficiency (↑ DF%, ↓RV%) with dobutamine stress ↑ mean ED KE of all flow components with dobutamine stress
Stoll et al. (76)	45 controls	Test-retest variability	Flow component **volumes** Flow component diastolic KE*	−	DF was the largest component, followed by RV, DEF and RI. DF had greatest mean ED KE, followed by RI, DEF, and RV.
Eriksson et al. (72)	12 controls 1 DCM patient	Normal blood flow	Flow component **volumes** Flow component **diastolic KE**	−	Reduced flow efficiency (↓DF%, ↑ RI + RV%) in DCM patient ↓mean ED KE of DF, ↑ overall diastolic KE of non-inflow volume (DEF + RV) in DCM patient
Bolger et al. (71)	17 controls 1 DCM patient	Normal blood flow	Flow component **volumes** Flow component **diastolic KE**	−	Reduced flow efficiency (↓DF%, ↑ RI + RV%) in DCM patient Similar DF diastolic KE loss in DCM patient, however greater loss of total inflow diastolic KE
**Cardiovascular disease**					
Stoll et al. (79)	64 HF patients 36 controls	HF (mixed etiology)	Flow component **volumes** Flow component diastolic KE*	−	Reduced flow efficiency (↓DF%, ↑ RI + RV%) in HF patients ↓mean ED and average KE of DF, ↑ mean ED KE of non-ejected volume (RI + RV) in HF patients.
Corrado et al. (73)	12 MI patients 10 controls	Post MI	Flow component **volumes** Flow component diastolic KE*	−	Reduced flow efficiency (↓DF%, ↑ RI, DEF and RV%) post ant. MI. No significant difference in average KE post ant. MI.
Karlsson et al. (78)	10 AF patients	Post cardioversion	Flow component **volumes** Flow component **diastolic KE**	−	Improved flow efficiency (↑ DF%, ↓RV%) post cardioversion. ↑ mean ED KE of DF, and ↓mean ED KE of RV post cardioversion.
Eriksson et al. (28)	6 controls 3 DCM patients	Semi-automatic analysis	Flow component **volumes**	−	The semi-automatic analysis approach used was accurate and had good reproducibility
Eriksson et al. (74)	10 DCM patients 10 controls	HF (DCM)	Flow component **volumes** Flow component diastolic KE*	−	Reduced flow efficiency (↓DF%, ↑ RI, DEF and RV%) in DCM. No significant difference in mean ED KE of DF, but ↑ ED KE of RI, DEF and RV in DCM.
Zajac et al. (80)	22 HF patients (50% with LBBB)	HF (mixed etiology) Dyssynchrony	Flow component **volumes** Flow component diastolic KE*	−	No significant difference in LVEDV ratio in patients with LBBB. ↓mean ED KE of Direct Flow in patients with LBBB.
Svalbring et al. (75)	26 IHD patients 10 controls	LV remodeling and dysfunction	Flow component **volumes** Flow component diastolic KE*	−	Reduced flow efficiency (↓DF%, ↑ RI + RV%) with increased LV volumes. ↓mean ED KE of Direct Flow, ↑ mean ED KE of non-ejected volume (RI + RV) with increased LV volumes.

DCM, dilated cardiomyopathy; DF, direct flow; DEF, delayed ejection flow; HF, heart failure; IHD, ischemic heart disease; KE, kinetic energy; LBBB, left bundle branch block; LV, left ventricle; MI, myocardial infarction; RI, retained inflow; RV, residual volume. *Indexed to flow component volume.

**TABLE 6:** Cardiac states and associated left ventricular diastolic flow features on 4D flow CMR.

	Physiological processes	Global flow analysis	Flow component analysis	Vortex flow analysis
**LV remodeling** (physiological)				
Athletes	↑ LVEDV ↑ LV mass	↑ peak E wave KE (33)		Preserved vortex formation Preserved vortex diastolic KE (33)
Advanced age	↑ LV stiffness ↓ LV compliance ↑ LVEDP	↓peak E wave KE (27, 30) ↑ peak A wave KE (25)		Reduced number and velocity of diastolic vortices
**LV remodeling** (pathological)				
Non-dilated post MI, cardiac amyloidosis, COPD	↑ LV stiffness ↓ LV compliance ↑ LVEDP	↓peak E wave KE (28)	Reduced flow efficiency (↓ DF, ↑ non-ejected volume) Reduced DF ED KE (73, 75)	↓ Early diastolic vortex KE ↓ Early diastolic vorticity (63, 67, 68)
Dilated DCM, post MI	↑ LVEDV ↑ LVEDP MV annular dilatation	↓ Peak E wave KE (32, 34) ↓ ED and mean diastolic KE (36) ↑ Turbulent KE (42)	Reduced flow efficiency (↓ DF, ↑ non-ejected volume) Reduced DF ED KE (74, 75, 79)	↑ Vortex mixing ratio (70) ↓ Proportion of diastolic KE (63)
LV dyssynchrony LBBB	Abnormal septal motion, incomplete LV filling	↑ Turbulent KE (42) ↑ Transverse forces (50)	Reduced DF ED KE (80)	−

COPD, chronic obstructive pulmonary disease; DCM, dilated cardiomyopathy; DF, direct flow; ED, end diastolic; KE, kinetic energy; LV, left ventricle; LVEDP, left ventricular end diastolic pressure; LVEDV, left ventricular end diastolic volume; MI, myocardial infarction; MV, mitral valve.

## Global flow analysis

### Left ventricular diastolic kinetic energy

As the LV actively expands in diastole and ‘sucks’ blood in from the left atrium, it confers kinetic energy and pressure to the blood. Based on Newton’s second Law of Motion, blood flow kinetic energy (KE) can be calculated using the equation:


$$\text{KE }(\text{μJ})=\text{1}/\text{2}\times \text{Mass}\times {\text{Velocity}}^{\text{2}}$$

(Where mass = mean density of blood (1060 g/mm^3^) × voxel volume).

Kinetic energy can be computed directly for the velocity in every voxel throughout the cardiac cycle and is typically summed over the whole left ventricle and indexed against left ventricular end diastolic volume (LVEDV) or stroke volume (SV) to negate the influence of heart size.

The conservation or loss of kinetic energy during diastole (diastolic KE) is thought to be a more reliable and direct marker of diastolic work than other parameters such as mitral inflow and myocardial velocity (27). Moreover, semi-automatic methods can now obtain KE values with a high degree of reproducibility and accuracy (27–29), and they have been proven to correlate with mitral inflow and annular velocity (27, 30).

In normal hearts there are 3 KE peaks (25, 31, 32), corresponding to the velocity peaks on trans-mitral Doppler echocardiography. These occur in mid-systole, early diastole (also referred to as the ‘E wave,’ representing rapid ventricular filling) and end/late diastole (also referred to as the ‘A wave,’ corresponding to atrial systole). In the LV, early diastole generates the highest KE peak—a reminder that ventricular relaxation is an active process (27, 32). In contrast, the highest peak in the right ventricle (RV) occurs during systole, suggesting that LV filling (diastole) may require greater myocardial work than systole, in contrast to RV filling (25, 33).

Steding-Ehrenborg et al. (33) found that athletes were able to generate higher diastolic KE peaks compared with controls despite no difference in mean diastolic KE. They, as well as others (31), found that the main determinant of the early diastolic KE peak was LV mass. Therefore, the greater the amount of healthy myocardium, the greater the strength of active diastolic relaxation and by extension LV diastolic KE. As of yet, LV diastolic KE has not been studied in patients with pathological hypertrophy such as seen in hypertrophic cardiomyopathy (HCM), which may involve a different relationship to diastolic KE.

**FIGURE 2:**
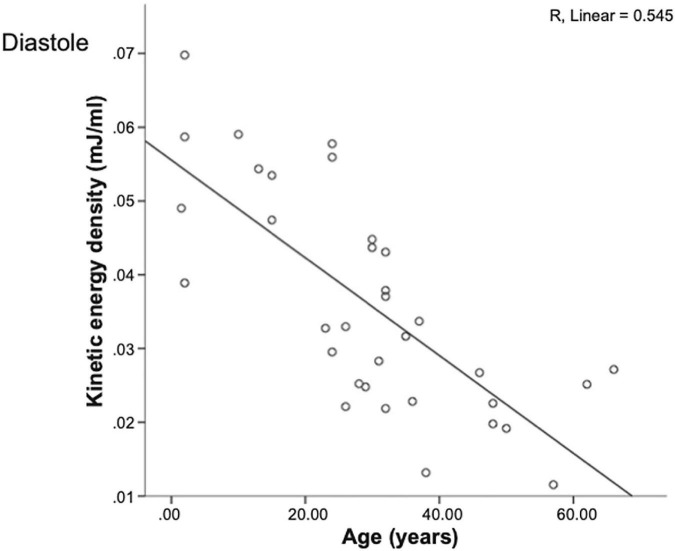
Peak left ventricular KE/ml in early diastole for healthy individuals with age (30). Early diastolic peak left ventricular KE/ml (generated during active relaxation) declines with increasing age.

Several studies have assessed the effects of age on diastolic KE. Early diastolic peak KE declines with age (27, 30) (Figure 2) with values in the elderly comparable to patient with LV impairment (30). This may be explained by worsening LV compliance and increased stiffness with age. In a study by Crandon et al. (27), there was both a decrease in early diastolic kinetic energy and compensatory increase in late diastolic KE with age, resulting in a reduced KE E/A ratio mirroring changes seen with LVDD in Doppler measurements of the E/A ratio.

**FIGURE 3:**
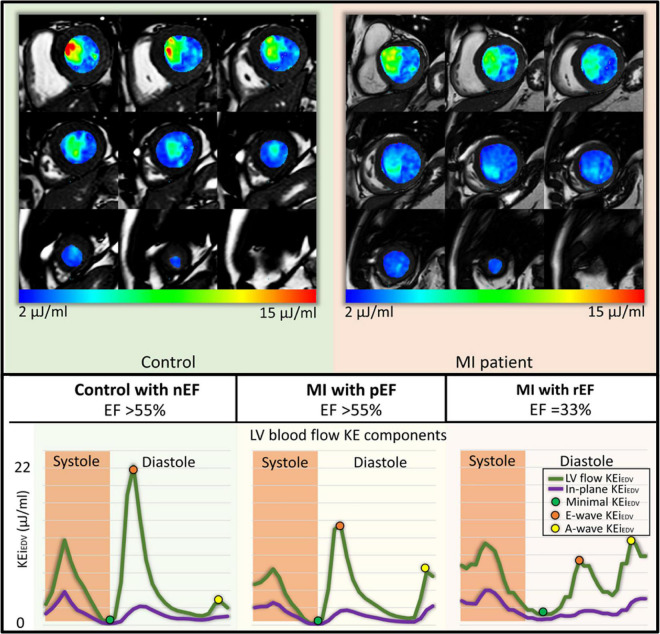
Left ventricular kinetic energy maps in a control and an MI patient **(Top)**. Kinetic energy curves in a control and two MI patients with preserved left ventricular ejection fraction (pEF) and reduced left ventricular ejection fraction (rEF) showing reduced peak E-wave KE **(Bottom)** (32).

Several studies have also assessed diastolic KE in heart failure patients (Table 1) (30, 31, 34). Left ventricular systolic dysfunction (LVSD) is associated with a reduction in both systolic and diastolic KE. Garg et al. (34) compared KE parameters (indexed to LVEDV) of patients with significant LVSD post myocardial infarction (MI) against healthy controls. Patients demonstrated a reduction in average KE as well as peak early diastolic KE values (Figure 3). Of note, an earlier study by Garg et al. (32) found that MI patients with preserved systolic function also had reduced peak E wave (early) diastolic KE – early evidence of the diastolic dysfunction that can occur post MI. A significant reduction in peak E wave KE was also observed in patients with cardiac amyloidosis (35), a condition characterized by diastolic dysfunction and heart failure with preserved ejection fraction.

Only one study has examined diastolic KE in the context of valvular heart disease. Al-Wakeel et al. (36) compared patients with severe mitral regurgitation (MR)—pre and post mitral valve (MV) surgery—with health volunteers. Compared with volunteers, preoperative patients demonstrated significantly greater mean LV KE, as well as early and late diastolic peak KE, though not when indexed to LV blood volume. After surgery, the expected reduction in LV volume and stroke volume resulted in a significant reduction in mean LV KE, systolic KE, and early diastolic KE (comparable to controls), confirming previously described interdependence of diastolic KE on LV volume. In contrast late diastolic KE in patients did not decrease post-operatively relative to controls, an observation that may have been due to age differences between patient and control groups.

### Turbulent kinetic energy and viscous energy loss

Not all the energy from myocardial relaxation is transferred into blood flow kinetic energy. Viscous Energy Loss (EL) represents the energy lost to heat (due to friction of blood against the ventricular wall), and Turbulent Kinetic Energy (TKE) refers to energy dissipated into small turbulent eddies. Both parameters are novel markers of flow inefficiency but have so far mostly been used to study energy loss in the context of congenital heart disease (37, 38) and aortic valvulopathy (39, 40).

Energy loss has been examined in a single study focusing on LV diastolic function (41) (Table 2). In this study by Elbaz et al., patients with altered mitral valve morphology secondary to atrioventricular septal defect (AVSD) closure (and consequently abnormal diastolic vortex formation), had significantly greater diastolic energy loss compared with healthy volunteers.

**FIGURE 4:**
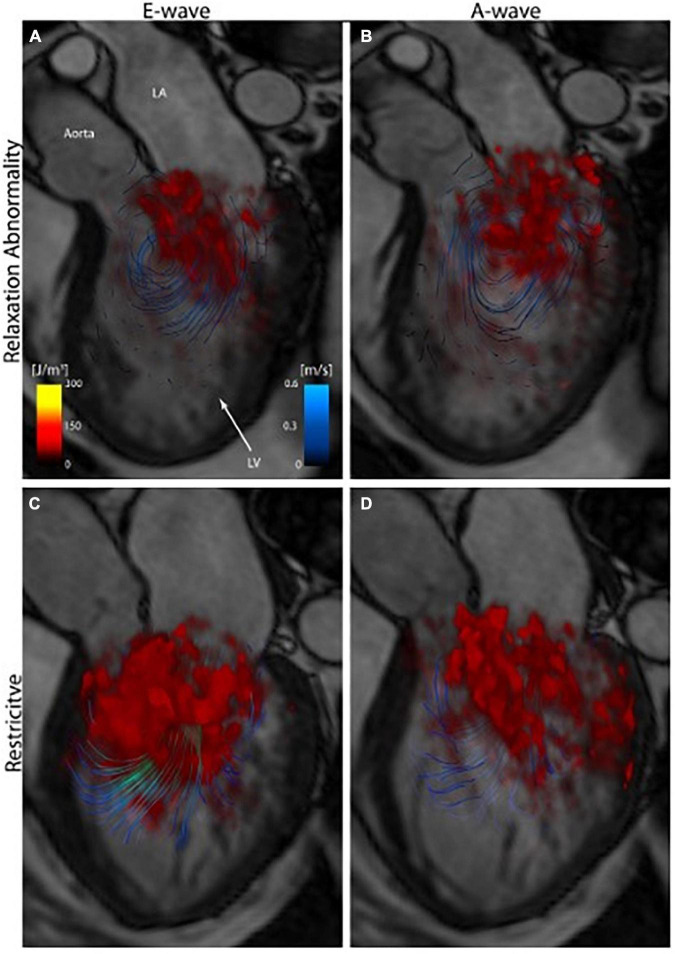
3D volume rendering of turbulent kinetic energy (TKE) (red) in early (E-wave) and late (A-wave) diastolic filling of patients with grade 1 diastolic dysfunction (relaxation abnormality)-top, and grade 3–4 (restrictive filling)-bottom, showing significantly greater turbulent kinetic energy with greater diastolic dysfunction (42).

Zajac et al. (42) compared the TKE of healthy volunteers and patients with varying degrees of LVDD secondary to dilated cardiomyopathy (DCM). They found that unlike normal controls, in DCM patients there was a trend toward higher LV TKE values with increasing LV volume (Figure 4). DCM patients also had significantly greater late diastolic TKE values which the authors hypothesized may reflect increased turbulence as inflowing blood encounters high LV end diastolic pressure (LVEDP) typically seen in LVDD.

## Relative pressure

Based on work by Ebbers et al. and others (43–45), 4D flow CMR has also enabled the measurement of relative intracardiac pressure differences by calculating pressure gradients from 3D velocity fields. In comparison to 2D PC-CMR, pressure gradients derived *via* this method are not reliant on correct identification of the direction/plane of maximum flow velocity and provide additional 3D spatial and temporal information. These relative pressure maps also present an attractive non-invasive alternative to catheter-based pressure measurements, which are susceptible to operator variability particularly with regards to catheter positioning. However, despite its promising potential as a non-invasive means of measuring intracardiac pressure derangements seen in diastolic dysfunction, relative pressure has yet to be studied in this condition or indeed compared against catheter measurements. Furthermore, current sequences add significantly to scanning time, and there is still much research in progress to refine the computational approach used to derive relative pressure maps from 3D flow velocity data (44, 46, 47).

## Hemodynamic force

Another novel 4D flow CMR marker, hemodynamic force (the force exerted by intraventricular blood flow on the myocardium) can also be derived from intraventricular pressure gradients (48, 49). Changes in the direction and magnitude of hemodynamic forces can reflect derangements in blood flow caused by impaired diastolic filling. Both Eriksson et al. (50) and Arvidsson et al. (51) (Table 2) showed that patients with heart failure with reduced ejection fraction with and without dyssynchrony experienced a significant reduction of normal diastolic hemodynamic forces along the long axis plane (along main direction of blood flow) and an increase in hemodynamic forces along the short axis or transverse plane (orthogonal to main direction of blood flow), indicative of impaired relaxation. Further study and refinement of this promising technique is required however, as to our knowledge only two 4D flow CMR studies have used hemodynamic force analysis in patients with heart failure with preserved ejection fraction, and they have produced some conflicting results [e.g., base to apical hemodynamic forces were not consistently abnormal in previous studies (52, 53)].

## Vortex flow analysis

**FIGURE 5:**
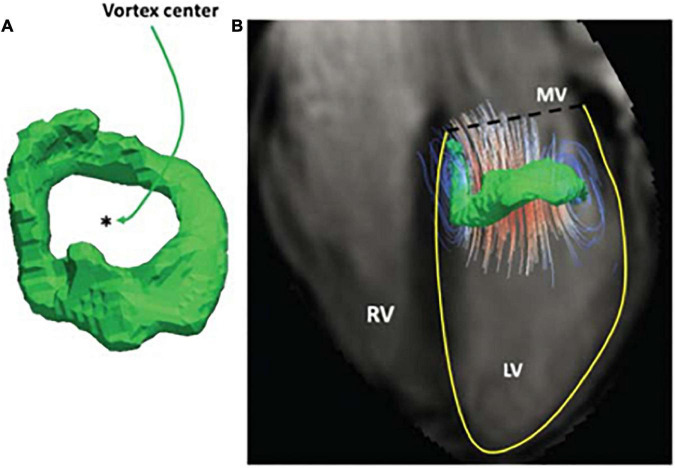
Left ventricular diastolic vortex ring in a healthy volunteer. Example of early diastolic vortex ring **(A)**, Streamlines superimposed on a vortex in a four-chamber view **(B)** (41).

As the left atrium empties into the LV and blood passes through the distal tips of the mitral valve, two ring-shaped (or ‘toroidal’) vortices are formed during early and late diastole (Figure 5). Although our understanding of the complex flow dynamics of blood remains incomplete, it is thought that LV vortex formation plays an important role in energy conservation, the redirection of blood flow and closure of the mitral valve leaflets (56, 57). Abnormalities in vortex formation have therefore been studied to provide insight into LV diastolic function.

Vortex assessment using 4D flow CMR is a relatively recent development, echocardiography studies have previously characterized vortex formation and have demonstrated that vortex malformation is associated with energy loss and diastolic dysfunction (58, 59). Early 4D flow CMR studies of LV diastolic vortices employed various complex visualization and analysis techniques (including Lambda2, Q criterion and Langragian coherent structures) to assess vortex dimensions, and to a lesser extent vortex vorticity and kinetic energy (Table 3). Additionally, 4D flow CMR imaging of pulmonary arterial flow has shown that the existence and relative duration of pulmonary vortices can be used as markers of pulmonary hypertension (60, 61), correlating well with invasive mean pulmonary arterial pressure (mPAP) measurements (61).

## Vortex dimensions

In normal physiology, the early diastolic vortex ring is smaller, more circular, and pulls blood toward the apex before dissipating at diastasis. A subsequent larger late diastolic vortex ring then forms which helps direct blood toward the LVOT (26, 62, 63). Vortex formation and size is closely linked with LV form and function (64). Vortex shape is to a significant extent determined by shape of mitral inflow (62), and therefore structural MV abnormalities may affect vortex formation and energy efficiency of blood flow. In a study comparing patients post AVSD repair to controls, Elbaz et al. (41) showed that altered diastolic vortex formation was associated with increased viscous energy loss. Ventricular and mitral annular dilatation also affect vortex formation. Suwa et al. (65) found that in patients with severe LV dysfunction and dilatation, diastolic vortices were more apically located, larger and more spherical. Even though vortices are larger in dilated ventricles, Töger et al. show that such vortices may make up a smaller proportion of LV volume compared to healthy controls (66). Even in the absence of significant LV dilatation, subtle changes in vortex dimensions may be seen in diseased hearts. Krauter et al. (63) compared 10 ischemic heart disease (IHD) patients to 10 controls, and showed that despite no significant difference in LVEDV, SV or ejection fraction, in IHD patients early diastolic vortices were more elliptical, and contained significantly lower absolute and relative (to vortex ring volume) kinetic energy.

## Vortex diastolic kinetic energy

A substantial proportion of the kinetic energy of diastolic blood flow is carried within the vortex, with Steding-Ehrenborg et al. (33) suggesting that as much as 70% of the total diastolic KE can be found within vortices. Therefore, reductions in vortex diastolic KE may indicate impaired LV diastolic function. Indeed, Kanski et al. (31) found that in patients with heart failure a smaller proportion of diastolic KE was found inside the vortex ring compared with healthy controls. In Krauter et al.’s study (63), patients with chronic IHD had reduced vortex diastolic KE compared with controls, despite no significant difference in LV volumes.

## Vorticity

**FIGURE 6:**
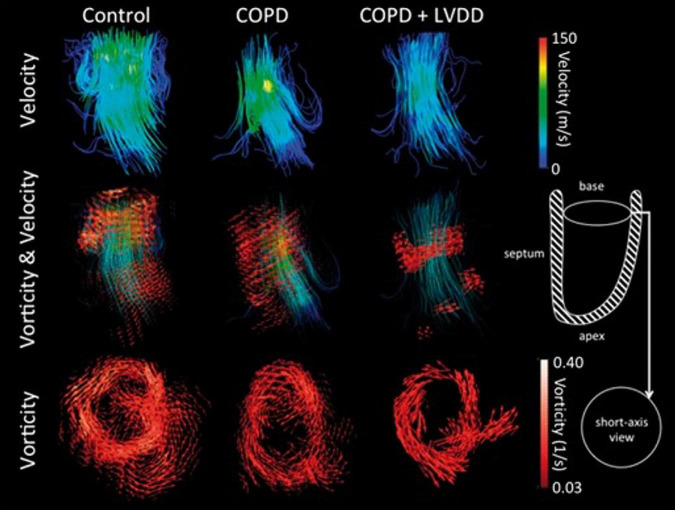
Streamline visualization with velocity color coding of diastolic flow in controls, COPD patients with and without left ventricular diastolic dysfunction (LVDD) **(Top row)**. Streamline visualization of diastolic flow with superimposed vorticity vector fields **(Middle row)**. Vorticity vector fields in all three groups, showing a loss of vorticity in both COPD with and without LVDD **(Bottom row)** (68).

Vorticity is a measure of the local rotation of fluid particles within a fluid as they travel through its main flow. Greater vorticity during ventricular inflow—which is dominated by a large ring vortex—is associated with conservation of kinetic energy leading to more efficient flow (29). This rotational property of the vortex has been shown to be a marker of diastolic function in several studies (41, 67, 68). Schäfer et al. (68) demonstrated that peak early diastolic vorticity was significantly reduced in patients with chronic obstructive pulmonary disease (COPD) with and without confirmed LVDD on echocardiography (Figure 6), suggesting that vorticity may be a more sensitive marker of diastolic dysfunction. Krauter et al. (63) similarly found reduced vorticity in chronic IHD patients compared with controls. In that study, vorticity correlated strongly with transmitral velocities measured with 2D PC-CMR. In another study by Schäfer et al. (67) examining vorticity in pulmonary hypertension patients, early diastolic vorticity correlated significantly with mitral annular velocities (septal and lateral e’) and E/A ratio—further indication that vorticity has potential as a measure of LV diastolic function.

## Flow component analysis

**FIGURE 7:**
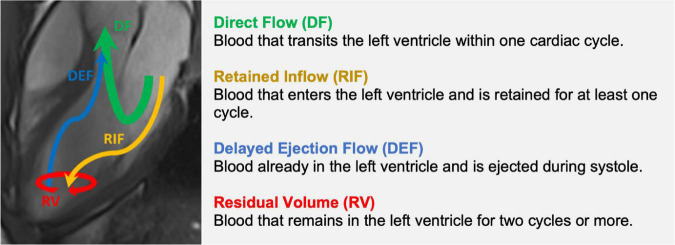
Constituent functional flow components of left ventricular blood volume.

First described by Bolger et al. (71) using particle trace analysis to visualize 3D blood flow, LVEDV can be separated into four functional flow components. These four components are Direct Flow (DF)—the most efficient component of ventricular blood which transits the heart in one cardiac cycle, Retained Inflow (RIF)—blood that enters the LV during diastole but is retained for at least one cycle, Delayed Ejection Flow (DEF)—blood already in the LV during diastole and which leaves during systole, and Residual Volume (RV)—blood that remains in the LV for at least two cycles (Figure 7). DF and RIF enter the ventricle during diastole and together may be referred to as *inflow*. *Outflow* consists of DF and DEF, the two components that leave the ventricle during systole. RIF and RV remain in the ventricle during systole, making up the *Non-ejected Volume*.

In normal hearts, DF is the largest component by proportion of LVEDV (∼35–40%), followed by RV (∼25–30%), with the remaining volume shared equally between RIF and DEF (Table 4).

## Flow component relative volume

The relative volume of the flow components reflects flow distribution within the ventricle and can be used as a measure of blood flow efficiency, with reduced relative proportions of DF and RV corresponding to a reduction in systolic but also diastolic function. This is because diastole is integral to creating favorable conditions for maximum DF and thereby flow efficiency. By the end of diastole, DF retains the greatest amount of kinetic energy of all the flow components (71) and is optimally positioned in terms of its angle and distance to the LVOT (72). Flow components (in particular DF) therefore reflect diastolic-systolic coupling and their relative volumes can be utilized as a useful marker of LVDD.

Table 4 summarizes the studies that describe flow components as a percentage of LVEDV in health controls. Most studies published demonstrate consistency in the proportions of the four functional flow components measured on 4D flow CMR. The two outliers were Bolger et al. (71) and Corrado et al. (73), with differences seen potentially due to disparate methodologies used in processing of 4D flow datasets.

Several studies have shown alterations in flow component relative volumes between patient groups and under certain conditions (Table 5).

**FIGURE 8:**
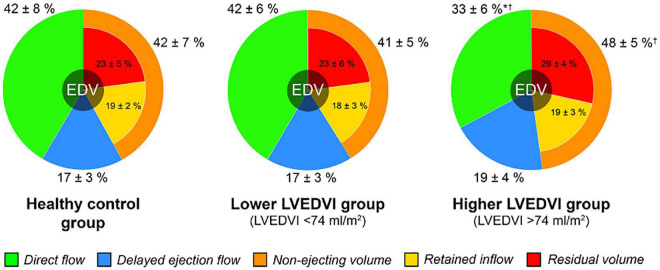
Left ventricular blood flow component distribution in healthy controls and in chronic ischemic heart disease patient subgroups (stratified by LVEDV index) (75).

In three different studies, Eriksson et al. (28, 72, 74) compared controls to dilated cardiomyopathy patients. They found that in heart failure patients, as the LV dilates, the proportion of non-ejected components increase at the expense of Direct Flow, which is diminished. Svalbring et al. (75) found a similar pattern in patients with mild ischemic cardiomyopathy with preserved systolic function, suggesting that blood flow component analysis may detect even subtle or subclinical abnormalities in LV remodeling (Figure 8).

Another study which points to the sensitivity of blood flow component analysis is Karlsson et al. (78). In this study, the authors were able to show that patients with atrial fibrillation who underwent cardioversion gained significant improvement in blood flow efficiency (increased DF, reduced RV) within 4 weeks of return to sinus rhythm.

Sundin et al. (77) studied changes in blood flow components during dobutamine stress testing in 12 healthy controls and found a similar improvement in blood flow efficiency, with a substantial improvement in DF and reduction in RV compared to rest.

## Flow component diastolic kinetic energy

Alongside assessing flow component volumes, 4D flow CMR techniques can also be used to measure the kinetic energy of individual components. The sum of the diastolic KE of the four flow components are equal to LV global flow diastolic KE. Several studies have shown a direct correlation between the flow component volume and flow component diastolic KE. This is unsurprising given that mass (volume) is a central component of KE. Consequently, component KE normalized to volume (i.e., KE/ml) has been reported by many as this provides incremental value (Table 5).

Studies of component diastolic KE have found that an increase in Direct Flow proportion was accompanied by an increase in DF end diastolic KE and a reduction in that of the residual volume end diastolic KE (77, 78), and that the converse relationship held true where there was reduction in DF proportion (72, 75, 79). The most comprehensive study highlighting the potential clinical utility of flow component diastolic KE was carried out by Stoll et al. (79). In this study, 64 heart failure patients (mixed etiology of DCM and ischemic cardiomyopathy) were compared with normal controls. In addition to significantly lower DF proportions, mean end-diastolic and average diastolic KE in heart failure patients, Stoll et al. found that derangements in diastolic KE values correlated with brain natriuretic peptide (BNP) levels, New York Heart Association (NYHA) classification, functional capacity as well as myocardial cellular energetics. Flow component relative volumes and diastolic KE could therefore become sensitive tools in the early detection and prognostication of LVDD in patients.

Lastly, the impact of dyssynchrony on flow component diastolic KE has also been studied. Zajac et al. (80) compared heart failure patients (mixed etiology) with and without left bundle branch block (LBBB) and found that despite no change in flow component volume, the early diastolic KE of the DF component was lower in patients with LBBB than those without. This likely reflects the inherent inefficiency of abnormal and dyssynchronous LV relaxation. This study also introduces the concept of flow component diastolic KE as a novel predictor of cardiac resynchronization therapy (CRT) response.

## Validation against established measures of left ventricular diastolic function

Few studies have compared novel 4D flow diastolic parameters against conventional echocardiographic indices of LV diastolic function—Doppler transmitral, mitral annular and tricuspid regurgitation velocities. The only study that compared diastolic KE was from Al-Wakeel et al. (36) and showed that diastolic KE E/A ratio correlated with Doppler E/A ratio—a predictable correlation which does not further the use of other 4D flow parameters highlighted here. Furthermore, this correlation was only significant in postoperative patients. In Zajac et al. (42), TKE correlated with echo-derived peak late diastolic (a wave) velocity. Lastly, Schäfer et al. (67), showed that 4D flow E and A wave vorticity correlated with multiple echo-based diastolic parameters including the Doppler E/A ratio. To our knowledge, there are currently no studies that have directly compared 4D diastolic parameters against invasive measurements of left ventricular filling pressures.

## Technical limitations

As a relatively new technology, 4D flow CMR is not yet widely accessible, and expertise are not widespread (81). FDA approved and CE marked 4D flow CMR sequences are now available on all modern MRI systems, but an acquisition with respiratory motion compensation and adequate temporal and spatial resolution may take up to 15 minutes (82). Moreover, temporal and spatial resolution may be insufficient for certain 4D flow parameters such as wall shear stress and pulse wave velocity (81).

Acquisition time can be reduced by eliminating respiratory motion compensation, but this can significantly impact image quality (81). Self-gating techniques are in development which may help negate the effects of respiratory motion (83). Several approaches to the reduce the acquisition time have been proposed, of which some may reach clinical application soon.

In 4D flow CMR, the normal practice of having a single velocity encoding range (or VENC) can result in an insufficient velocity to noise ratio (VNR) in regions with low velocities. Techniques that enable multi-VENC measurements allow for a better VNR over the whole velocity range, but at the cost of a longer acquisition time (84).

Prior to 4D flow data analysis, pre-processing to correct for phase offset errors such as eddy current effects and velocity aliasing is necessary. For this, several commercial FDA approved and CE marked software solution are available, but adequate technical expertise is still required as optimal strategies may vary according to MR system, 4D flow CMR sequence and application (81). Adequate routines for quality assurance and validation are recommended (81).

Data analysis and 4D flow visualization techniques, like acquisition and processing methods, may differ from center to center, and not all parameters can be measured in all centers. As a result, a variety of different parameters have been used across studies, meaning that results are often not directly comparable, and some studies have had conflicting results (71, 73).

Lastly, 4D flow CMR generates huge data sets and therefore adequate data management, and storage infrastructure are essential to be able to process 4D flow data.

## Future direction

Further, more comprehensive, validation of 4D parameters against existing non-invasive and invasive markers is required, and ideally linked to clinical outcomes to determine the true utility of these techniques. Testing at different field strengths is also required, as is cross-vendor standardization of acquisition, processing, and analysis techniques, before 4D flow can be more widely used clinically for assessment of diastolic function—as outlined in the 2015 Society for Cardiovascular Magnetic Resonance (SCMR) consensus statement (81).

Research into the assessment of LV diastolic function using 4D flow CMR has so far consisted almost entirely of small single center studies. Larger multicenter and multimodality studies are necessary for comprehensive assessment of HFpEF (and other cardiac conditions involving LVDD), and more definitive comparisons of the performance of novel 4D parameters against contemporary diagnostic and prognostic markers of disease.

## Conclusion

Early studies of LVDD using time resolved 3-dimensional velocity mapping acquired with 4D flow CMR have contributed to our understanding of complex physiological and pathophysiological processes such as energy loss, vortex formation and functional blood flow components (Table 6). Novel 4D flow parameters such as global flow diastolic KE, vorticity, and flow component relative volumes have shown significant promise as sensitive markers of early and pre-clinical LVDD, and some have shown a correlation with standard 2D parameters of LV diastolic function. These novel markers could play a future role in refining the diagnosis of HFpEF and in the monitoring of LVDD in many cardiac conditions—from valve disease to ischemic heart disease—especially in patients with poor acoustic windows (e.g., obesity, COPD), and could allow CMR to better challenge echocardiography as the non-invasive modality of choice for the assessment of LV diastolic function.

## Author contributions

ZA, SM, C-JC, TE, and BR made substantial contributions to the concept and design of the manuscript. All authors including SN made substantial contributions to the drafting and revision of the manuscript. All authors have approved the submitted version.

## Conflict of interest

The authors declare that the research was conducted in the absence of any commercial or financial relationships that could be construed as a potential conflict of interest.

## Publisher’s note

All claims expressed in this article are solely those of the authors and do not necessarily represent those of their affiliated organizations, or those of the publisher, the editors and the reviewers. Any product that may be evaluated in this article, or claim that may be made by its manufacturer, is not guaranteed or endorsed by the publisher.
